# Comorbid Profile Rather than Age Determines Hip Fracture Mortality in a Nonagenarian Population

**DOI:** 10.1007/s11420-015-9435-y

**Published:** 2015-07-31

**Authors:** Adam Graver, Sara Merwin, Lewis Collins, Nina Kohn, Ariel Goldman

**Affiliations:** 1 Department of Orthopaedic Surgery, Long Island Jewish Medical Center, 270-05 76th Avenue, New Hyde Park, NY 11040 USA; 2 Hofstra North Shore-LIJ School of Medicine, 500 Hofstra University, Hempstead, NY 11549 USA; 3 North Shore-LIJ Medical Group, University Orthopaedic Associates, 611 Northern Blvd Suite 200, Great Neck, NY 11021 USA; 4 Biostatistics Unit, Feinstein Institute for Medical Research, 350 Community Drive, Manhasset, NY 11030 USA; 5 North Shore University Hospital, 300 Community Drive, Manhasset, NY 11030 USA

**Keywords:** fragility fractures, orthopedic, postoperative, geriatrics, comorbidity, mortality

## Abstract

**Background:**

In light of poor outcomes with nonoperative management of hip fractures, orthopedic surgeons are faced with difficult decisions about which patients are too ill or too old for surgical treatment.

**Questions/Purposes:**

This study sought to investigate if patients over 90 years had different preoperative laboratory, clinical, and injury characteristics than younger patients with the same injury. We compared our cohort with previously published data. We wished to identify if there were pre-injury risk factors associated with 30-day mortality, which could be modified to enhance postoperative outcomes.

**Methods:**

This is a retrospective review of 198 operatively managed hip fractures in patients 75 years or older. We collected data on demographics, select preoperative laboratory values, injury type, comorbidities, and 30-day mortality.

**Results:**

Eleven (5.6%) of the cohort died within 30 days of surgery, 6.3% in the younger group, and 3.7% in the older group; the difference was not statistically significant. For baseline characteristics, there was no difference between the age groups for pre-injury comorbidities, hemoglobin, serum albumin, BUN, prevalence of UTI, or fracture type. A total of 67 (35.8%) patients had evidence of UTI on admission.

**Conclusions:**

These findings reveal that in our dichotomized cohort, pre-injury characteristics were similar and age alone was not an independent predictor of mortality. These data may inform decision-making for orthopedic surgeons and the medical providers who consult to optimize these patients for surgery. We identified high rates of UTI in both age groups, a potentially remediable factor to optimize outcomes in hip fracture surgery in elderly patients.

**Electronic supplementary material:**

The online version of this article (doi:10.1007/s11420-015-9435-y) contains supplementary material, which is available to authorized users.

Original Release Date: July 30, 2015Expiration Date: July 30, 2016

## Overview

As the population ages and longevity increases, there is a growing incidence of what demographers term “old old” patients incurring hip fractures. In light of poor outcomes with nonoperative management, orthopedic surgeons must decide which patients are too ill, too frail, or too old for surgical treatment. This controversy faces orthopedic surgeons every day and around the world. The preoperative factors associated with overall increased mortality are well studied and have historically included older age, poor baseline mental and ambulatory status, and multiple chronic diseases [1, 8, 20].

The impetus to conduct this investigation was based on an anecdotal observation by the senior author that nonagenarians appeared to have similar 30-day outcomes as his younger (yet elderly) hip fracture patients. To understand this phenomenon and to test its assumption, several lines of inquiry were initiated to investigate if our nonagenarians who sustained hip fractures had attributes which were special or distinct from other previously studied samples, if our treatment strategy was unique, or if there were innate protective factors in individuals whose survival reached a tenth decade. To address these questions, we chose to examine the conventional predictors of mortality after hip fracture and employed a post-hoc risk schema which had previously been applied in this fashion. Multiple authors have described the increasing odds of 30-day mortality in older groups of patients, specifically patients over 90 [18, 23].

Given the observation that age did not seem to be a risk factor for mortality in our cohort, our study attempts to answer the following three questions: 1) do patients 90 years and older in our cohort have different preoperative laboratory, clinical, and injury characteristics than younger patients with the same injury? 2) What potentially modifiable contributing risk factors (demographic, laboratory findings, and clinical) were associated with 30-day mortality and were there differences between our older and younger cohorts for this outcome?

## Learning Objectives

Hospital for Special Surgery professional education activities are intended to improve knowledge, competence, and performance of our learners and to lead to better patient care. At the conclusion of the activity, the participant should be able to:

- Identify comorbid predictors, other than 90+ years of age, such as low scoring on mental status tests, age, body mass index (BMI), and history of malignancy, for patients who suffered from hip fractures within 30 days.
- Implement appropriate treatments to practice for at-risk hip lfracture patients, including counseling of patients and families on postoperative risks and whether or not surgery is an appropriate treatment.

## Target Audience

This activity is targeted at orthopaedic surgeons, primary care physicians, rheumatologists, gerontologists, specialty physicians, physician assistants, residents, fellows and medical students.

## Accreditation

Hospital for Special Surgery is accredited by the Accreditation Council for Continuing Medical Education to provide continuing medical education for physicians.

## Credit Designation

Hospital for Special Surgery designates this journal-based CME activity for a maximum of 1.0 *AMA PRA Category 1 Credit(s)*™. Physicians should claim only the credit commensurate with the extent of their participation in the activity.

## Commercial Support

This journal-based activity did not receive commercial support.

## Faculty Disclosure

n accordance with the Accreditation Council for Continuing Medical Education’s Standards for Commercial Support, all CME providers are required to disclose to the activity audience the relevant financial relationships of the planners, teachers, and authors involved in the development of CME content. An individual has a relevant financial relationship if he or she has a financial relationship in any amount occurring in the last 12 months with a commercial interest whose products or services are discussed in the CME activity content over which the individual has control.

It is the policy of the Hospital for Special Surgery to disclose all financial relationships that planners, teachers, and authors have with commercial interests. Relationship information appears below:

**Activity Directors Disclosure:**

**Ariel Goldman, MD** has disclosed no relevant financial relationships.

**Adam Graver, MD** has disclosed no relevant financial relationships.

**Planning Committee Disclosure:**

**Charles N. Cornell, MD** has disclosed no relevant financial relationships.

**Natanya Gayle, MPH** has disclosed no relevant financial relationships. 

**Ariel Goldman, MD** has disclosed no relevant financial relationships.

**Adam Graver, MD** has disclosed no relevant financial relationships.

**Antonia Costello, LCSW** has disclosed no relevant financial relationships.

**Lewis Collins, OPA** has disclosed no relevant financial relationships.

**Sara Merwin, MPH** has disclosed no relevant financial relationships.

**Nina Kohn, MBA, MA** has disclosed no relevant financial relationships.

**OCME/CME Committee Disclosure:**

Hospital for Special Surgery Office of CME Staff and CME Committee members have no relevant financial relationships to disclose regarding this activity.

**Activity Faculty**

Activity Directors:

Ariel Goldman, MD

Orthopaedic Trauma Surgeon

North Shore University Hospital,

Long Island Jewish Medical Center (NSLIJ)

Orthopaedic Surgery

Great Neck, NY

Adam Graver, MD

Orthopaedic Surgery Resident

North Shore LIJ

New Hyde Park, NY

**Planning Committee**

Lewis Collins, OPA

Orthopaedic Surgery Assistant

University Orthopaedic Associates

Great Neck, NY

Charles N. Cornell, MD

Clinical Director of Orthopaedic Surgery

Attending Orthopaedic Surgeon

Hospital for Special Surgery

Professor of Clinical Orthopaedic Surgery

Weill Cornell Medical College

New York, NY

Antonia Costello, LCSW

Professional Education Program Coordinator

Education & Academic Affairs

Hospital for Special Surgery

New York, NY

Ariel Goldman, MD

Orthopaedic Trauma Surgeon

North Shore University Hospital,

Long Island Jewish Medical Center (NSLIJ)

Orthopaedic Surgery

Great Neck, NY

Adam Graver, MD

Orthopaedic Surgery Resident

North Shore LIJ

New Hyde Park, NY

Nina Kohn, MBA, MA

Senior Biostatistician

Biostatistics Unit, Feinstein Institute for Medical Research

Manhasset, NY

Sara Merwin, MPH

Research Associate

Department of Orthopaedic Surgery, Long Island Jewish Medical Center and North Shore University Hospital

New Hyde Park, NY

Natanya Gayle, MPHManaging Editor, HSS Journal New York, NY

**TECHNICAL REQUIREMENTS**

**Windows**

- Microsoft Windows XP, Windows Server 2003, Windows Vista, Windows Server 2008, Windows 7 operating system
- Microsoft Internet Explorer 6.0 SP1 or later, Firefox 2.0 or later, or Google Chrome 1.0 (Chrome is only supported on Mediasite version 5.0.3 and later) Web browser
- Windows Media Player 9 or later
- For Firefox and Chrome playback, Microsoft Silverlight 1.0 or later (viewers are prompted to install this plug-in when attempting to view a presentation)
- Broadband internet connection (256 kbps or more)

**Mac – Requires Mediasite 4.3 and later:**

- Mac OS X 10.4.8 or later operating system
- Safari 2.0.4 or later or Firefox 2.0 or later Web browser
- Microsoft Silverlight 1.0 or later (viewers are prompted to install this plug-in when attempting to view a presentation).
- Broadband internet connection (256 kbps or more)

For more information, please visit Sonic Foundry http://www.sonicfoundry.com/contact/.

Please view our privacy policy http://www.hss.edu/notice-of-privacy-practices.asp.

**Instructions for Post-test, Course Evaluation and CME Credit:**

In order to earn CME credit, you must complete an online or print post-test and evaluation following the completion of this activity. There is a passing requirement of 100%. Once you complete the post test and subsequent evaluation, a certificate will be available for you to print.

For questions related to the post-test and subsequent evaluation, please contact the HSS Journal at gaylen@hss.edu or 646-797-8509.

*Option 1: Take the post-test on-line.*

1. Go to the HSS Journal homepage at www.springer.com/hss.
2. Click on ‘CME and Free-to-Access Articles’ tab.
3. Click on “Comorbid Profile Rather Than Age Determines Hip Fracture Mortality in a Nonagenarian Population” to view the full-text pdf article.
4. After you have reviewed the article click on ‘Complete the Current CME Test Online’ to register and complete the test.

*Option 2: Take the post-test via hard copy printed in the Journal*.

**You may complete the hard copy of the post-test. Please mail the test to:**

Journal CME Post-Test

Office of Continuing Medical Education

Education Division

535 East 70^th^ Street

New York, NY 10021

## Introduction

Hip fractures are a significant cause of morbidity, mortality, and health care consumption in the elderly, accounting for 258,000 hospital admissions per year in the USA [13]. Incidence increases with age and is principally in women [3, 8]. Although there is evidence that incidence of hip fracture is declining, prevalence of comorbidities among these patients is increasing [4]. Worldwide total number of hip fractures has been estimated to exceed 6 million by 2050 [16]. As the population ages and longevity increases, there is a growing incidence of what demographers term “old old” patients incurring hip fractures. In light of poor outcomes with nonoperative management, orthopedic surgeons must decide which patients are too ill, too frail, or too old for surgical treatment. This controversy faces orthopedic surgeons every day and around the world. The preoperative factors associated with overall increased mortality are well studied and have historically included older age, poor baseline mental and ambulatory status, and multiple chronic diseases [1, 8, 20]. Independent predictors of 30-day mortality include advanced age, male gender, several comorbidities, poor mini-mental admission test score, hemoglobin concentration less than 10 g/dL, institutionalization, and malignant disease [9, 19, 21, 24]. Preoperative risk assessment tools such as the Nottingham hip fracture score, POSSUM, and American Society of Anesthesiologists (ASA) and NSQIP have been developed to help guide treatment decisions [17, 19, 21, 23]. The presence of two or more comorbid conditions has been identified as a harbinger of poor outcomes [18, 19, 24]. Previously and extensively used to predict mortality in medical patients, the Charlson comorbidity index (CCI) uses a weighted score for 21 comorbidities associated with mortality [6, 22]. In a cohort of hip fracture patients, a Charlson score of greater than 6 has been shown to double the odds ratio for mortality within 30 days [18].

The impetus to conduct this investigation was based on an anecdotal observation by the senior author that nonagenarians appeared to have similar 30-day outcomes as his younger (yet elderly) hip fracture patients. To understand this phenomenon and to test its assumption, several lines of inquiry were initiated to investigate if our nonagenarians who sustained hip fractures had attributes which were special or distinct from other previously studied samples, if our treatment strategy was unique, or if there were innate protective factors in individuals whose survival reached a tenth decade. To address these questions, we chose to examine the conventional predictors of mortality after hip fracture and employed a post-hoc risk schema which had previously been applied in this fashion. Multiple authors have described the increasing odds of 30-day mortality in older groups of patients, specifically patients over 90 [18, 24].

Given the observation that age did not seem to be a risk factor for mortality in our cohort, our study attempts to answer the following three questions: 1) Do patients 90 years and older in our cohort have different preoperative laboratory, clinical, and injury characteristics than younger patients with the same injury? 2) How does our cohort compare with US and international data; are our results generalizable or is our cohort unique [5, 15, 18, 19, 24]? 3) What potentially modifiable contributing risk factors (demographic, laboratory findings, and clinical) were associated with 30-day mortality and were there differences between our older and younger cohorts for this outcome?

## Patients and Methods

**Fig. 1:**
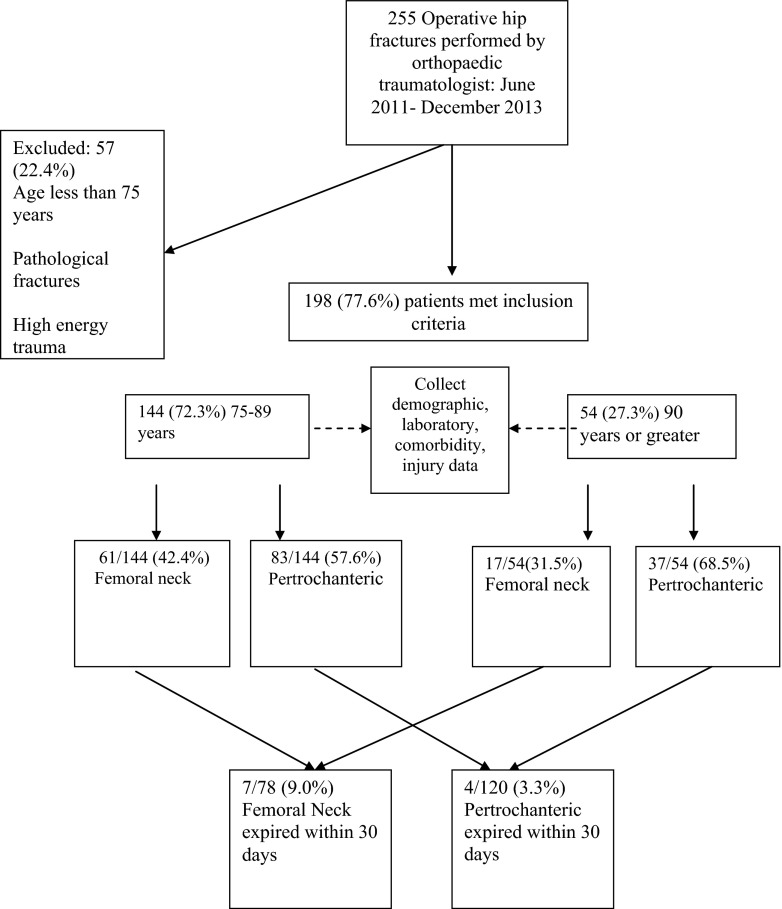
Study flow diagram.

This retrospective case control study was conducted at two tertiary care, teaching hospitals on Long Island, NY. All operative hip fracture patients meeting inclusion criteria from June 2011 to December 2013 were included in a retrospective review of cases from one fellowship-trained orthopedic trauma surgeon’s hip fracture database. Patients included in the study were geriatric hip fracture patients treated operatively by the senior author. Pertrochanteric fractures were treated with dynamic hip screw, or cephalomedullary nail and femoral neck fractures were treated with open reduction internal fixation (ORIF), hemiarthroplasty, or total hip arthroplasty. Patients were excluded if they were less than 75 years of age, suffered high-energy trauma, and had nonoperatively managed hip fractures or pathologic fractures. All patients were assessed and treated using an algorithmic approach (Fig. 1).

Baseline characteristics that were recorded included age, gender, fracture type, calculation of pre-injury comorbid disease states, admission hemoglobin, BUN, creatinine, albumin, and the presence of urinary tract infection (UTI), defined by the presence of positive urine culture or leukocyte esterase, or nitrates, on urine analysis. All laboratory results collected were from a single preoperative value at time of admission. We defined injury types as follows: femoral neck (AO/OTA 31-B) and pertrochanteric (AO/OTA 31-A). To categorize comorbidities, we used the Charlson comorbidity index, a widely employed, validated instrument for predicting mortality using a composite score in which weights are assigned to commonly encountered conditions [6].

The medical records of 255 consecutive patients within the surgeon’s database were examined. Fifty-seven (22.4%) of patients were excluded. All of those excluded were under 75 years of age. Among the patients 75 years or older, there were none with high-energy trauma or pathological fractures. One hundred and ninety-eight (77.6%) out of 255 total consecutive patients with hip fractures in the surgeon’s database met inclusion criteria during the study period. Of these, 54 (27.3%) were 90 years or older. The mean age for the older group was 93.2 (SD ±3.3), the median age in the over 90 group was 92. Of the patients over 90 years, 16 or 29.6% were 95 or older, with the oldest patient 104. For the 75–89-year-old group, the mean age was 83.2 (SD ±3.7), median age was 83.5. For the entire group, the mean age was 85.9 years (SD ±5.8), median age was 85 years. Male patients constituted 30.8% of the cohort, 31.3% of the younger group, and 29.6% in the older group.

Our primary outcome measure was 30-day mortality. The patients were stratified into two groups based on age at the time of admission. The older group consisted of patients older than 90 years, and the younger group was patients aged 75 to 89 years. We chose the cutpoint of 75 for the younger group to mirror a similar range of the ages we observed in the older group (90 to 104). We examined the same set of preoperative factors (demographics, laboratory values, comorbidities, and injury type) to correlate and compare previously identified predictors of 30-day mortality [5, 15, 18, 19, 21, 24]. Since increasing age has previously been shown to be an independent risk factor for mortality, this investigation focused on 30-day outcomes.

Clinical, surgical, and demographic data were abstracted from the surgeon’s billing database and electronic medical record systems employed by the two institutions (Sunrise Clinical Systems and AllScripts). We used the social security death index to confirm mortality within 30 days of surgery.

For the purpose of statistical analysis, descriptive data are presented as frequencies and percentages for categorical variables, and means with standard deviations and medians for continuous variables. The association between age group (75–89 years, ≥90 years) and categorical factors was examined using the Fisher’s exact test. The association between age group and continuous factors was examined using the Mann–Whitney test. The association between categorical factors and 30-day mortality was examined using the Fisher’s exact test. The association between continuous factors and 30-day mortality was examined using the Mann–Whitney test. Results were considered statistically significant at the *p* < 0.05 (two-sided) significance level. Data were analyzed using SAS Version 9.3 (SAS Institute Inc., Cary, NC) Table 1.

**Table 1:** Preoperative characteristics of 155 operatively managed hip fracture patients

	75–89 years *N* = 144	≥90 years *N* = 54	*p* value
Mean age, years ± SD	83.2 ± 3.7	93.2 ± 3.3	
Median, years	83.5	92.0	
Sex			
Male, *n* (%)	45 (31.25)	16 (29.63)	
Female, *n* (%)	99 (68.75)	38 (70.37)	ns
Fracture type			
Femoral neck, *n* (%)	61 (42.36)	17 (31.48)	
Trochanteric, *n* (%)	83 (57.64)	37 (68.52)	ns
Preop hgb, g/dL			
Mean ± SD	12.5 ± 1.6	12.2 ± 1.5	
Median	12.6	12.1	ns
Preop creatinine, mg/dL			
Mean ± SD	1.2 ± 1.0	1.2 ± 0.5	
Median	0.9	1.1	0.043
Preop BUN, g/dL^a^			
Mean ± SD	25.7 (12.9)	27.2 (12.3)	
Median	22.0	24.0	ns
Preop albumin, g/dL^b^			
Mean ± SD	4.0 (0.4)	3.9 (0.3)	
Median	4.0	3.9	ns
Preop UTI (leukest on UA)^c^			
Positive, *n* (%)	50 (36.76)	17 (33.33)	
Negative, *n* (%)	86 (63.24)	34 (66.67)	ns
CCI score			
Mean (SD)	1.7 (2.1)	1.4 (1.0)	
Median	1.0	1.0	ns

^a^Sample size for BUN age 75–89: 142, age ≥90: 54

^b^Sample size for albumin age 75–89: 131, age ≥90: 50

^c^Sample size for UTI age 75–89: 136, age ≥90: 51

## Results

No significant differences between the age groups were found with regard to gender, preoperative hemoglobin, serum albumin, BUN, the presence of UTI, or fracture type. Serum creatinine was marginally significant (Table 2). There were no differences between the two age groups for the individual Charlson comorbidities (Table 3). Sixty-nine (34.8%) of the combined cohort had two or more of the Charlson comorbid conditions included in the calculation of the index. The age groups did not differ on Charlson score. The mean Charlson score for the younger group was 1.7 (SD ±2.1) and 1.4 (SD ±1.0) for the older group. In the younger group, 15.3% had a Charlson score in the highest two categories (≥4) compared to 1.9% of the older group; no patients in the older group had Charlson score of 6 or greater points (Table 2). When both age groups were combined, patients with femoral neck fracture had a higher Charlson comorbidity index than subjects with pertrochanteric fractures (*p* < 0.015).

**Table 2:** Frequencies of Charlson comorbidities in operative managed hip fracture patients by age cohort

	75–89 years	≥90 years	*p* value
	*N* = 144	*N* = 54	
	*N* (%)	*N* (%)	
MI	11 (7.64)	2 (3.70)	ns
CHF	12 (8.33)	10 (18.52)	ns
CVD	19 (13.19)	9 (16.67)	ns
PVD	23 (15.97)	6 (11.11)	ns
Dementia	27 (18.75)	17 (31.48)	ns
Peptic ulcer	2 (1.39)	1 (1.85)	ns
Mild liver disease	0 (0.00)	1 (1.85)	ns
DM	19 (13.19)	5 (9.26)	ns
Connective tissue	1 (0.69)	0 (0.00)	ns
COPD	15 (10.42)	3 (5.56)	ns
Hemiplegia	6 (4.17)	0 (0.00)	ns
Mod–severe renal disease	13 (9.03)	4 (7.41)	ns
DM w/ end organ damage	3 (2.08)	0 (0.00)	ns
Tumor w/i 5 years	25 (17.36)	5 (9.26)	ns
Leukemia	1 (0.69)	1 (1.85)	ns
Lymphoma	2 (1.39)	0 (0.00)	ns
Mod–severe liver dz	0 (0.00)	0 (0.00)	
Metastatic solid tumor	3 (2.08)	0 (0.00)	ns
AIDS	0 (0.00)	0 (0.00)

**Table 3:** Thirty-day mortality in operatively managed hip fracture patients

	Alive *N* = 187	Deceased *N* = 11	*p* value
Younger, *n* (%)	135 (93.75)	9 (6.25)	
Older, *n* (%)	52 (96.30)	2 (3.70)	ns
Male, *n* (%)	55 (90.16)	6 (9.84)	
Female, *n* (%)	132 (96.35)	5 (3.65)	ns
Fracture type			
Femoral neck	71 (91.03)	7 (8.97)	
Trochanteric	116 (96.67)	4 (3.33)	ns
CCI score			
Mean ± SD	1.6 ± 1.9	1.8 ± 1.2	
Median	1.0	2.0	ns
Preop hgb (g/dL)			
Mean SD	12.4 ± 1.6	12.6 ± 1.7	
Median	12.4	12.8	ns
Preop creatinine (mg/dL)			
Mean, SD	1.2 ± 0.7	1.8 ± 2.6	
Median	1.0	0.9	ns
Preop BUN (units)^a^			
Mean, SD	25.7 ± 12.4	32.8 ± 16.2	
Median	23.0	33.0	ns
Preop albumin (units)^b^			
Mean, SD	3.9 ± 0.4	3.7 ± 0.5	
Median	4.0	3.8	ns
Preop UTI (leukest on UA)^c^			
Positive *n* (%)	61 (91.04)	6 (8.96)	
Negative *n* (%)	116 (96.67)	4 (3.33)	ns

^a^Sample size for BUN alive: 185, deceased: 11

^b^Sample size for albumin alive: 171, deceased: 10

^c^Sample size for UTI alive: 177, deceased: 10

For the outcome 30-day mortality, the 95% exact confidence interval was 13.25 to 18.2% for the difference (2.55%) between the mortality rates. The characteristics of the patients who survived the initial 30-day postoperative period and those who expired were compared. There were no differences for gender, age, type of injury, comorbidity, or preoperative laboratory values. Eleven or 5.6% of the cohort died within 30 days of surgery: 6.3% in the younger group and 3.7% in the older group (Table 4).

**Table 4:** Charlson comorbidity index operatively managed hip fracture patients—comparison between our study cohort and the Kirkland cohort

	0 pts *n* (%)	1 pt *n* (%)	2–3 pts *n* (%)	4–5 pts *n* (%)	6+ pts *n* (%)	*p* value
75–89 years	48 (33.33)	40 (27.78)	34 (23.61)	12 (8.33)	10 (6.94)	
≥90 years	12 (22.22)	19 (35.19)	22 (40.74)	1 (1.85)	0 (0.00)	0.0113
Total	60 (30.30)	59 (29.80)	56 (28.28)	13 (6.57)	10 (5.05)	
Kirkland (64–106)	114 (23.5)	99 (20.4)	131 (27.0)	54 (11.1)	87 (17.9)	N/A

Among the patients screened on admission for urinary tract infections, 67 (36.8% of the younger group and 33.3% of the older group) had evidence of UTI on admission as evidenced by positive leukocyte esterase on urine analysis.

## Discussion

In our cohort of operatively managed hip fractures, we had the anecdotal observation that patients over the age of 90 had no worse 30-day perioperative mortality compared with our younger patients, suggesting age is not an independent risk factor for mortality in operative hip fracture patients. Our analysis confirmed this observation; we observed no differences in preoperative characteristics between the age groups.

Our study was subject to several limitations: the retrospective nature of the study precludes the ability to make causal inferences. Further, observational studies may be subject to unknown biases, including selection bias. Our small sample size and low event frequency leave the study relatively underpowered since it was based on available charts and therefore not subject to a post-hoc power analysis. Our results may be the reflection of the low mortality “signal” and therefore may not represent the truth. Further investigation as this cohort grows in size will be useful to see if the effect holds true. Our findings may have poor external validity as our patient population may be unique, with overall good access to health care and very few uninsured.

We noted no clinically relevant differences between nonagenarians and the age stratum below for preoperative and injury characteristics. Although event frequency was low, there was no difference between age groups for 30-day mortality (Table 2). A plausible explanation is that individuals who survive past 90 years of age have “favorable” phenotypes with cardiovascular protective factors [2, 11, 12]. Among the nonagenarians in our cohort, the cardiovascular profile, as categorized by the Charlson index, was comparable to the younger group. However, overall, our nonagenerians appear to be healthier than the younger group; 15.3% of the younger group had a Charlson score of 4 or more compared to 1.9% of the older group. We contrasted the distribution of the Charlson comorbidities scores in our cohort with a similar study of hip fracture patients [18] (Table 5).

We compared our patients’ characteristics and outcomes with other hip fracture cohorts in the USA, UK, Europe, and Asia. In contrast to several published reports, age alone did not significantly predict 30-day mortality after hip fracture surgery. Few studies have specifically focused on hip fracture characteristics and outcomes in nonagenarians with the exception of one Japanese investigation in which higher 1-year mortality was observed in patients above the age of 89 but no differences between the groups in preoperative health as quantified using the ASA grading system [15]. Our cohort had a higher proportion of patients 90 years or older (27.3%), was apparently healthier, and had a higher proportion of males than similar investigations.

**Table 5:** Characteristics and outcomes: comparison between our study and other hip fracture studies

	Our study	Kadowaki	Castronuovo	Kirkland	Maxwell	Roche
Country, year	US, 2013	Japan, 2012	Italy, 2011	US, 2011	UK, 2008	UK, 2005
Total *n*	198	186	6896	485	4967	2448
Study design	Retrospective chart review	Retrospective chart review	Prospective cohort: administrative data registry	Retrospective chart review	Prospective cohort	Prospective observational
Mean age (year) SD	85.9 (5.8)	84	n/a	82.8 (7.8)	80	82
Median age, (range)	85 (75–104)	(65–105)	83 (65–106)	83 (64–106)	(17–102)	(60–103)
Female gender, *n* (%)	137 (69.19)	151 (81.2)	5387 (78)	356 (73.4)	76.8%	1955 (80)
30-day mortality, *n* (%)	11 (5.56)	3 (1.6)	6.3%^a^	53 (10.9) 36 (9.7) over 75 years	390 (7.9)	231 (9.6)
90 years or more, *n* (%)	54 (27.3)	50 (26.9)	n/a^c^	96 (19.8)	n/a	465 (19)
Type of fracture						
Femoral neck	78 (39.39)	58 (31)	6378 (92.5%)	n/a	4967 (99.9) 0	57% Intracapsular, 43% extracapsular
Trochanteric	120 (60.61)	128 (69)				
% Conservative	Excluded	Excluded	16.2%	7.3%	Excluded	Excluded^d^
Preop risk scoring method	Charlson	ASA	Modified Charlson by Deyo (for ICD9)	Charlson	Nottingham	
Patients with 2 or more comorbid conditions, *n* (%)	69 (34.85)	51 (27)^b^	(9.1)	56.1%	1.155 (23.3)	576 (24)

*n*/*a* data not available

^a^Included patients managed nonoperatively

^b^Solely corresponds to the amount of patients with 3 or more comorbidities, study does not include amount of patients with only 2 comorbidities

^c^2,726 (39.5%) greater than 84 years

^d^Excluded 358 patients including all those aged <60; those with periprosthetic fractures, pathological fractures; fractures treated without surgery; patients who died before surgery

This phenomenon may be explained by an aggressive treatment strategy by the senior author. Advances in anesthesia techniques now make it possible for patients with multiple medical comorbidities to achieve favorable outcomes and the ability to regain pre-fracture level of function.

Compared to other investigations into short-term mortality after surgery for hip fracture, our 30-day rates were slightly lower (5. 6% versus a range from 6.3 to 9.7%) with the exception of a Japanese investigation in which 98% survived to 30 days [5, 15, 18, 19, 24].

Previous reports identified greater risk of femoral neck fractures with increasing age, whereas we did not find that age predicted the type of hip fracture. In our cohort, patients with femoral neck fractures had a higher Charlson score than those with pertrochanteric fractures (*p* < 0.01), indicating poorer overall health. This is in contrast to previous investigations in which poor health status predisposed patients to intertrochanteric fractures [10].

Overall, in contrast to the US study which used the Charlson comorbidity index, there was not a relationship between age or high Charlson score and 30-day mortality; our patients had lower average Charlson score and fewer patients in the highest Charlson group [18] (Table 5).

Other reports found that patients with a diagnosis of dementia were at increased risk of mortality [14, 18]. In our cohort, dementia was not associated with 30-day mortality. However, 31.5% of patients over the age of 90 had a diagnosis of dementia at baseline, compared with 18.7% in the younger group. This difference was not statistically significant.

It is unclear why our patient population had certain preoperative characteristics and results that were different than other studies. One possible explanation is that at baseline, our population has high socioeconomic status, good access to health care, positive health behaviors, and reasonably favorable nutritional status. Moreover, a strict “door to table” protocol was observed; patients undergoing operative management of hip fracture at our two institutions were taken to the OR within 48 h of admission, a measure which has been demonstrated to improve overall mortality [25, 26]. Our findings may be reflective of the standardized technique employed by a single surgeon.

We detected unexpectedly high rates of apparent urinary tract infections on admission laboratory results in both age groups. Although not associated with 30-day mortality in this investigation, the presence of infection should be considered as a potential source of perioperative morbidity.

In conclusion, in our investigation of elderly patients with operatively managed hip fractures, there were no differences in preoperative comorbidities and clinically relevant laboratory values between the age groups. Additionally, there was no difference in the outcome of 30-day mortality. Our results indicate that patients over 90 years old presenting for operative management for hip fracture are similar to those in our younger cohort. Although not associated with poorer outcomes in this study, the high rates of apparent UTI provide an opportunity to intervene to mitigate the risk of potential complications. Similarly, metabolic disarray and other factors may signal cause for concern. Notably, the findings from this investigation suggest that age alone should not impact surgical decision-making for patients with hip fractures. Other markers of frailty and its correlates should be considered as they may imperial survival in elderly patients with operative management of hip fracture. The findings in this study may inform decision-making for orthopedic surgeons and for the medical providers who consult to clear these patients for surgery to repair hip fractures as well as for patient and family counseling.

### Electronic supplementary material

Below is the link to the electronic supplementary material.

ESM 1 (PDF 1224 kb)

ESM 2 (PDF 1224 kb)

ESM 3 (PDF 1224 kb)

ESM 4 (PDF 1224 kb)

ESM 5 (PDF 1224 kb)

## CME ACTIVITY: Comorbid profile rather than age determines hip fracture mortality in a nonagenarian population

CME QUESTIONS:

1. True or False: Incidence of hip fractures among very elderly individuals is decreasing.
2. Which of the following factors have not previously been associated with mortality after hip fracture surgery?
3. Older age
4. Female gender
5. Poor mental status at baseline
6. Presence of several comorbid diseases
7. True or False: In the present study, age over 90 years was associated with 30-day mortality.
8. Which of the following abnormal preoperative laboratory have been associated with poor outcomes in hip fracture patients in previous studies:
9. Hemoglobin, BUN, HgbA1C, Creatinine, Albumin
10. BUN, Creatinine
11. Hemoglobin, HgbA1C, Albumin
12. BUN, Creatinine
13. Albumin
14. Which of the following are WELL-VALIDATED risk scoring tools to evaluate mortality?
15. ASA
16. Charlson comorbidity index
17. NSQIP
18. A, B, C
19. A,B
20. 30-day mortality in operative hip fracture patients has been found to range approximately:
  - 0–3%
  - 4–6%
  - 6–10%
  - Over 25%
21. The primary outcome measure in the present study was:
  - Comorbidity profile
  - 30-day mortality
  - Intraoperative complication rate
  - 90-day mortality
22. Previous reports indicate _________ risk of femoral neck fractures with increasing age and _______ incidence of intertrochanteric fractures with poorer health status.
  - Similar, Increased
  - Similar, Similar
  - Decreased, Similar
  - Increased, Increased
  - Increased, Similar
23. True or False: The present study found a significant difference in preoperative injury characteristics between patients over 90 and a younger age group:
24. Significant differences were found in the present study with regard to the following:
  - Gender
  - Pre-operative hemoglobin
  - Serum albumin
  - Fracture type
  - None of the above
